# Stability and Infectivity of SARS-CoV-2 and Viral RNA in Water, Commercial Beverages, and Bodily Fluids

**DOI:** 10.3389/fmicb.2021.667956

**Published:** 2021-05-05

**Authors:** Mizuki Fukuta, Zhan Qiu Mao, Kouichi Morita, Meng Ling Moi

**Affiliations:** ^1^Graduate School of Biomedical Sciences, Nagasaki University, Nagasaki, Japan; ^2^Program for Nurturing Global Leaders in Tropical and Emerging Communicable Diseases, Nagasaki University, Nagasaki, Japan; ^3^WHO Global Reference Laboratory for COVID-19, WHO Collaborating Center for Reference and Research on Tropical and Emerging Virus Diseases, Institute of Tropical Medicine, Nagasaki University, Nagasaki, Japan

**Keywords:** SARS-CoV-2, virus stability, commercial beverages, body fluids, food hygiene and safety

## Abstract

The stability and infectivity of severe acute respiratory syndrome coronavirus 2 (SARS-CoV-2) in liquid samples are of great concern to virus transmission via common beverages and sewage water. Here, we investigated the stability of SARS-CoV-2 in 32 liquids including common beverages, bodily fluids, and commonly used viral transport media. Our results showed that the infectious virus could be recovered up to 77-days from common beverages including milk and water. Viral RNA could be detected at high levels in all samples up to 28-days, indicating that while viral RNA demonstrates higher stability than infectivity, viral RNA levels do not reflect the infectious capability of SARS-CoV-2. These results indicate that SARS-CoV-2 is highly stable in optimal conditions and a sufficient control measure is needed in reducing the risk of exposure and controlling and preventing future outbreaks.

## Introduction

Severe acute respiratory syndrome coronavirus 2 (SARS-CoV-2) viral RNA has been detected in surfaces and environment and surfaces due to contact with carrier (Kampf et al., 2020; van Doremalen et al., 2020). SARS-CoV-2 can survive in suspension and in aerosols in the environment for a limited time and the virus is very stable in favorable environments (Chin et al., 2020). At refrigeration temperatures (4°C), SARS-CoV-1 remains infectious up to 9 days (Rabenau et al., 2005). In a transportation medium, there was a limited reduction in infectious SARS-CoV-2 held at 4°C for up to 14 days (Chin et al., 2020). In this context, SARS-CoV-2 remained infectious for 3 weeks in spiked pieces of refrigerated chicken, pork, and salmon (Fisher et al., 2020). In industrial production, SARS-CoV-2 RNA has been detected in dairy products, including milk powder and ice cream, leading to disruptions in the food supply chain (Liew, 2021). While the persistence of viral RNA on surfaces and in food could contribute to potential positive test results, a robust risk evaluation should include an assay that is capable of differentiating between infectious and non-infectious virions. To date, there have been limited studies on viral infectivity in common beverages and bodily fluids. In this context, the evaluation of virus stability together with infectivity in commonly available beverages are of high importance to the risk assessment of outbreak control and prevention.

A current tool for the identification and assessment of SARS-CoV-2 includes reverse-transcriptase PCR (RT-PCR) for the detection of the virus genome and virus isolation. RT-PCR provides rapid assessment but offers no information on virus infectivity and is occasionally prone to false-positive results (Surkova et al., 2020; Wikramaratna et al., 2020). While laborious compared to the RT-PCR method, virus titration by using cell culture is the most direct method for the evaluation of virus infectivity. In this study, the infectivity of SARS-CoV-2 in 32 commercial beverage and bodily fluids was quantitated by using RT-PCR and a conventional plaque assay by cell culture; tested for infectivity up to 77 days after incubation, the aim was to evaluate the stability and infectivity of SARS-CoV-2 in liquid suspension.

## Materials and Methods

### Virus and Cell Lines

SARS-CoV-2 (TY-WK-521/2020 strain) was propagated on Vero 9013 cells at 37°C in 5% CO_2_ for up to 6 days. Vero 9013 cells (Japan Health Science Research Resources TBankT) were cultured as previously described in Eagle’s Minimum Essential Medium (EMEM, Sigma, St. Louis, MO) supplemented with heat-inactivated 10% fetal calf serum (FCS, Sigma) without antibiotics at 37°C in 5% CO_2_ (Balingit et al., 2020). Commercial beverages were obtained from local stores. Pooled saliva and urine used as body fluids samples were obtained from Innovative Research, Inc. (Novi, MI, United States). At set time points, samples were collected for the infectivity test by plaque assay and genomic viral RNA quantitation by quantitative real-time RT-PCR (qRT-PCR) (Corman et al., 2020; Le et al., 2020).

### Plaque Assay

A plaque assay was performed to determine the levels of infectious virus particles (Cooper, 1962). Infection assay using SARS-CoV-2 was performed in the BSL-3 facility at the Institute of Tropical Medicine, Nagasaki University. In this study, 50 μL of virus culture (2 × 10^6^ PFU/mL) was inoculated into 950 μL of the liquid sample (final virus concentration was 1 × 10^5^ PFU/mL) and incubated at 4°C. In clinical samples, the infectious virus was isolated from the nasopharyngeal swabs of COVID 19 patients at titers between 10^5^ PFU/mL to 10^7^PFU/mL (Lescure, 2020), suggesting that virus shedding occurs between these levels during natural infection. Thus, the liquid samples in this study were spiked at concentrations that show virus shedding during natural infection at a titer of 2 × 10^6^ PFU/mL of virus (final concentration: 1 × 10^5^ PFU/mL). Briefly, SARS-CoV-2 spiked liquid samples were incubated at 4°C were collected at each time point and serially diluted in the EMEM (dilution ratio of 1:10–1:10^3^). A total of 100 μL of serially diluted sample was inoculated onto monolayers of Vero cells in a 12-well plate. Next, the plates were incubated at 37°C in 5% CO_2_ up to 40 min, and, after incubation, 2 mL of overlay media [5 g of Methylcellulose (Wako Pure Chemical Industries Ltd., Osaka, Japan), 12 g of Avicel RC 591 (FMC Biopolymer, United States), and 9.4 g of EMEM powder were dissolved in 1 L of DDW] was added to each well. After 3 days post-infection, cells were fixed with 4% paraformaldehyde and stained with 0.25% crystal violet (Wako Pure Chemical Industries) (Balingit et al., 2020). The number of plaques was counted with the naked eye, and the viral titer was defined as plaque-forming units per milliliter (PFU/mL).

### Viral RNA Quantitation

Viral RNA was extracted from 100 μL of liquid samples using Quick Viral RNA kit (Zymo research, CA, United States), and the SARS-CoV-2 E gene was amplified by qRT-PCR (Fisher et al., 2020; Wikramaratna et al., 2020). The PCR master mix consisted of 2.5 μL of RNA, 5 μL of TaqMan Fast Virus 1-Step Master Mix (Applied Biosystems, CA, United States), 0.25 μL of 100 μM forward and reverse primer, 0.5 μL of 10 μM probe, and 9 μL nuclease-free water. Viral RNA was subjected to quantitative RT-PCR as previously described (Corman et al., 2020; Le et al., 2020). The viral RNA levels were defined as log 10 copies number/mL.

### Statistical Analysis

All virus titration assays (plaque titration and RT-qPCR) assays were performed in duplicates, and all experiments were at least performed twice. Data were analyzed by using GraphPad Prism version 6.07 (GraphPad software, La Jolla, CA, United States). For the determination of *P*-values of virus titers of the same samples, a paired Student’s *t*-test was used. Unpaired *t*-test was used for comparison of virus titers as determined by plaque assay and RT-PCR. A *P*-value of less than 0.05 (*P* < 0.05) is considered significant. The half-life value (day) of the infectious virus in each liquid sample was calculated using; t 1/2 = log_10_2/k, k = decay constant (Riddell et al., 2020).

## Results

A total of 32 types of liquid samples were used in this experiment. The spiked samples incubated at 4°C were collected at 13 incubation time points. Infectious virus and viral RNA were detected by plaque assay and qRT-PCR, respectively. The infectious virus value was represented as a plaque forming unit (PFU)/mL.

Infectious virus could be recovered between day 7 to day 35 from some beverages, including milk, fruit juices, alcoholic beverages (alcohol content < 16%), and water, and bodily fluids (saliva and urine, Table 1). Of the 32 samples tested, in four liquid samples, infectious virus particles could be detected up to day 77 of the incubation time point. Retention of viral infectivity varied according to the type of beverage and the incubation time (day 0, Table 1). There was a more than 10-fold reduction in the infectious titer in the 28 types of liquid samples tested. Overall, virus infectivity was reduced in most of the samples tested but remained infectious in 15/32 (46.9%), 14/32 (43.7%), and 11/32 (34.4%) of the samples tested at 14, 21, and 28 days, respectively. In this context, there was no significant reduction in SARS-CoV-2 infectious virus titers in common beverages, including milk and soy milk samples, up to day 28 of incubation (Table 1). In alcoholic beverage samples, infectious virus could be detected in three of the five spiked samples tested after 7 days of incubation. Viral RNA was detected in all of the samples tested, and the difference in the viral RNA levels between non-infectious and infectious samples was not significant by using the *t*-test (P _infectious vs.__non–__infectious_, day7 = 0.75). Viral RNA could still be detected at high levels in samples that did not demonstrate infectivity, concurring with that of previous studies that have shown that viral RNA levels do not reflect infectivity (Table 2). Between infectious and non-infectious samples, or according to the incubation period, viral RNA levels did not decrease significantly by the *t*-test (P _days__0 vs. 28_ = 0.33) and could be detected at high levels (>log10 10 genome copies/mL) at up to 1 month of incubation (Table 2). The half-life value of infectious SARS-CoV-2 in the water and milk group, bodily fluids and viral transport media were, however, had longer half-lives compared to other beverage groups (Table 3).

**TABLE 1:** Stability and infectivity of SARS-CoV-2 in different beverages and bodily fluids over an 11-week period.

	**Virus titer (log10 PFU/mL)**												
**Sample**	**Day 0**	**Day 3**	**Day 7**	**Day 14**	**Day 21**	**Day 28**	**Day 35**	**Day 42**	**Day 49**	**Day 56**	**Day 63**	**Day 70**	**Day 77**
**Group 1 water**													
Mineral water^a^	3.7 ± 0.2	3.5 ± 0.1	3.7 ± 0.1	3.6 ± 0.4	3.6 ± 0.1	3.1 ± 0.0	3.1 ± 0.0	ND	NT	NT	NT	NT	NT
Tap water^a^	4.6 ± 0.0	4.7 ± 0.1	4.8 ± 0.2	4.7 ± 0.0	4.3 ± 0.1	NT	3.9 ± 0.2	NT	3.5 ± 0.0	3.6 ± 0.0	NT	NT	NT
Distilled water	3.9 ± 0.1	3.4 ± 0.0	4.1 ± 0.2	4.1 ± 0.1	3.9 ± 0.2	3.6 ± 0.2	3.5 ± 0.0	3.4 ± 0.2	3.2 ± 0.0	3.4 ± 0.0	3.5 ± 0.0	3.0 ± 0.2	2.7 ± 0.1
**Group 2 milk**													
Milk	3.6 ± 0.1	3.1 ± 0.1	3.8 ± 0.1	3.7 ± 0.3	3.5 ± 0.1	3.3 ± 0.2	3.3 ± 0.0	3.1 ± 0.1	2.9 ± 0.1	3.2 ± 0.1	3.0 ± 0.2	2.2 ± 0.2	2.5 ± 0.2
Soy milk	3.5 ± 0.0	3.1 ± 0.1	4.4 ± 0.4	3.8 ± 0.0	4.0 ± 0.3	4.2 ± 0.5	2.9 ± 0.2	3.7 ± 0.2	2.7 ± 0.3	2.7 ± 0.1	2.9 ± 0.2	2.5 ± 0.0	2.4 ± 0.1
Yogurt drink	3.5 ± 0.0	2.9 ± 0.2	2.5 ± 0.2	ND	ND	ND	ND	NT	NT	NT	NT	NT	NT
Milk-coffee	3.2 ± 0.2	3.5 ± 0.0	2.9 ± 0.0	2.0^d^	2.2 ± 0.2	ND	ND	NT	NT	NT	NT	NT	NT
Cocoa	3.4 ± 0.2	3.6 ± 0.0	3.1 ± 0.2	2.6^d^	ND	ND	ND	NT	NT	NT	NT	NT	NT
**Group 3 juice/coffee**													
Apple juice	3.2 ± 0.9	3.7 ± 0.2	ND	ND	ND	ND	ND	NT	NT	NT	NT	NT	NT
Orange juice	3.3 ± 0.1	3.7 ± 0.1	3.3 ± 0.1	2.4^d^	ND	ND	ND	NT	NT	NT	NT	NT	NT
Tomato juice	3.7 ± 0.0	3.7 ± 0.0	3.5 ± 0.1	ND	ND	ND	ND	NT	NT	NT	NT	NT	NT
Flavored fruit juice	3.6 ± 0.1	3.4 ± 0.0	1.4 ± 1.7	ND	ND	ND	ND	NT	NT	NT	NT	NT	NT
Black coffee	3.0 ± 0.2	3.3 ± 0.2	2.8 ± 0.0	ND	ND	ND	ND	NT	NT	NT	NT	NT	NT
**Group 4 carbonated beverage**													
Cola	2.5 ± 0.2	2.3^d^	ND	ND	ND	ND	ND	NT	NT	NT	NT	NT	NT
Lemon-lime carbonated beverage	2.2 ± 0.3	ND	ND	ND	ND	ND	ND	NT	NT	NT	NT	NT	NT
**Group 5 tea**													
Green tea	2.7^d^	2.0^d^	ND	ND	ND	ND	ND	NT	NT	NT	NT	NT	NT
Black tea (Oolong tea)	ND^c^	ND	ND	ND	ND	ND	ND	NT	NT	NT	NT	NT	NT
Herbal tea (*Artemisia capillaris*)	ND	ND	ND	ND	ND	ND	ND	NT	NT	NT	NT	NT	NT
**Group 6 alcohol beverage**													
Beer, 5%^b^	3.6 ± 0.1	3.8 ± 0.1	3.5 ± 0.0	3.1 ± 0.1	3.1 ± 0.1	2.8 ± 0.3	2.3 ± 0.0	2.2 ± 0.3	2.0 ± 0.0	ND	NT	NT	NT
Sparkling sake, 5%^b^	3.3 ± 0.3	2.8 ± 0.1	ND	ND	ND	ND	ND	NT	NT	NT	NT	NT	NT
Carbonated distilled beverage (shochu), 9%^b^	3.4 ± 0.2	3.7 ± 0.0	3.9 ± 0.0	3.2 ± 0.0	3.1 ± 0.3	2.8 ± 0.1	ND	NT	NT	NT	NT	NT	NT
White wine, 11%^b^	ND	ND	ND	ND	ND	ND	ND	NT	NT	NT	NT	NT	NT
Japanese wine (sake), 15.6%^b^	3.4 ± 0.3	2.9 ± 0.0	3.1 ± 0.1	2.3^d^	2.7 ± 0.6	2.1 ± 0.2	ND	NT	NT	NT	NT	NT	NT
**Group 7 health beverage**													
Sports drink P	3.3 ± 0.2	2.6 ± 0.2	2.0^d^	ND	ND	ND	ND	NT	NT	NT	NT	NT	NT
Amino sports beverage	2.8 ± 0.2	ND	ND	ND	ND	ND	ND	NT	NT	NT	NT	NT	NT
Multivitamin beverage	3.3 ± 0.3	2.2 ± 0.2	2.0 ± 0.0	ND	ND	ND	ND	NT	NT	NT	NT	NT	NT
Energy tonic	ND	ND	ND	ND	ND	ND	ND	NT	NT	NT	NT	NT	NT
Vinegar beverage	3.8 ± 0.3	3.5 ± 0.1	2.8 ± 0.0	ND	ND	ND	ND	NT	NT	NT	NT	NT	NT
**Group 8 bodily fluid**													
Saliva^c^	NT^f^	3.9 ± 0.0	3.6 ± 0.0	3.2 ± 0.1	2.5 ± 0.3	2.0 ± 0.0	ND	NT	NT	NT	NT	NT	NT
Urine^c^	4.0 ± 0.2	3.6 ± 0.2	3.9 ± 0.1	3.7 ± 0.2	3.6 ± 0.1	3.1 ± 0.2	3.0 ± 0.1	NT	NT	NT	NT	NT	NT
**Group 9 viral transport media**													
PBS	3.9 ± 0.1	3.5 ± 0.1	4.2 ± 0.2	4.0 ± 0.2	3.0 ± 0.1	4.0 ± 0.1	3.3 ± 0.1	ND	NT	NT	NT	NT	NT
10% FBS/MEM	3.8 ± 0.1	3.5 ± 0.0	4.3 ± 0.3	3.9 ± 0.3	3.7 ± 0.1	3.6 ± 0.3	3.4 ± 0.4	3.2 ± 0.1	2.8 ± 0.0	3.0 ± 0.0	3.2 ± 0.2	2.7 ± 0.3	2.3 ± 0.0

*Viral titers are expressed as mean ± standard deviation log10 PFU/mL. Viral titers were titrated using Vero cells. All experiments were performed using at least two independent duplicates.*

*^*a*^Initial virus titer is higher due to differences in virus stock batch used in a spiked-tap water sample.*

*^b^Indicates alcohol content.*

*^c^Pooled saliva and urine were obtained from Innovative Research, Inc. (Novi, MI, United States).*

*^d^Only one replicate demonstrated plaques.*

*^e^ND below the detection limit. The detection limit of the assay was log10 2.0 PFU/mL.*

*^f^NT indicates not tested. FBS/MEM, 10% fetal bovine serum with minimum essential medium; PBS, phosphate-buffered saline.*

**TABLE 2:** SARS-CoV-2 genome levels in different beverages and bodily fluids as determined by real-time PCR.

	**Viral RNA titer (log10 genome copies/mL)**^a^				
**Sample**	**Day 0**	**Day 3**	**Day 7**	**Day 14**	**Day 28**
**Group 1 water**					
Mineral water	10.8 ± 6.8	11.0 ± 7.0	9.9 ± 6.8	10.1 ± 6.6	11.0 ± 6.1
Distilled water	11.0 ± 7.3	11.1 ± 7.5	11.3 ± 8.0	10.8 ± 6.4	10.8 ± 5.1
**Group 2 milk**					
Milk	10.9 ± 6.4	11.4 ± 7.6	9.8 ± 6.3	10.8 ± 5.7	10.5 ± 5.9
Soy milk	10.0 ± 6.3	10.3 ± 5.9	10.1 ± 5.9	10.0 ± 6.5	10.1 ± 5.6
Yogurt drink	10.3 ± 6.3	10.8 ± 6.0	10.1 ± 6.4	10.3 ± 6.1	10.6 ± 6.3
Milk-coffee	10.4 ± 6.0	10.9 ± 6.2	9.4 ± 5.6	9.0 ± 5.5	10.2 ± 6.1
Cocoa	9.3 ± 5.8	10.7 ± 6.7	9.2 ± 6.3	9.5 ± 6.1	10.3 ± 5.7
**Group 3 juice/coffee**					
Apple juice	10.4 ± 6.3	10.3 ± 5.3	10.2 ± 6.6	10.3 ± 5.4	10.6 ± 5.5
Orange juice	10.3 ± 7.0	10.8 ± 6.2	10.8 ± 6.1	10.8 ± 6.7	10.0 ± 6.1
Tomato juice	9.3 ± 5.6	10.0 ± 5.8	10.7 ± 6.6	10.7 ± 6.0	10.1 ± 5.4
Flavored fruit juice	11.1 ± 7.5	10.2 ± 5.2	10.2 ± 6.1	10.8 ± 5.9	10.3 ± 5.8
Black coffee	11.0 ± 7.3	11.5 ± 5.9	10.0 ± 6.2	10.8 ± 6.3	10.8 ± 6.0
**Group 4 carbonated beverage**					
Cola	10.4 ± 4.6	10.9 ± 4.4	11.1 ± 6.5	10.1 ± 5.5	10.7 ± 6.3
Lemon-lime carbonated beverage	10.6 ± 6.9	10.3 ± 6.1	9.6 ± 5.6	9.9 ± 5.9	10.9 ± 6.7
**Group 5 tea**					
Green tea	11.1 ± 7.2	11.2 ± 6.2	10.2 ± 6.6	10.8 ± 6.9	10.6 ± 5.8
Black tea (Oolong tea)	11.1 ± 6.7	11.6 ± 7.4	10.0 ± 4.9	10.9 ± 6.6	10.1 ± 5.4
Herbal tea (*Artemisia capillaris*)	11.2 ± 7.1	11.3 ± 6.0	10.9 ± 7.0	10.5 ± 5.5	10.1 ± 4.1
**Group 6 alcohol beverage**					
Beer, 5%^b^	11.4 ± 8.0	11.1 ± 7.3	10.7 ± 7.0	10.6 ± 6.0	10.8 ± 5.4
Sparkling sake, 5%^a^	10.2 ± 5.9	11.1 ± 6.0	10.9 ± 7.0	9.8 ± 4.9	10.9 ± 4.5
Carbonated distilled beverage (shochu), 9%^a^	10.6 ± 5.7	11.5 ± 6.9	11.2 ± 7.2	9.7 ± 5.4	10.8 ± 6.4
White wine, 11%^a^	10.9 ± 7.1	10.8 ± 6.8	9.6 ± 5.2	10.0 ± 5.5	10.6 ± 6.4
Japanese wine (sake), 15.6%^a^	10.8 ± 5.3	10.5 ± 6.6	9.7 ± 6.7	10.2 ± 6.2	10.4 ± 5.9
**Group 7 health beverage**					
Sports drink P	10.8 ± 6.6	11.5 ± 7.4	11.1 ± 7.5	10.9 ± 6.6	10.8 ± 6.1
Amino sports beverage	10.5 ± 6.4	11.0 ± 5.9	10.9 ± 6.9	10.5 ± 6.7	10.7 ± 5.8
Multivitamin beverage	10.0 ± 5.7	9.5 ± 4.7	9.9 ± 6.6	10.0 ± 4.7	9.3 ± 5.5
Energy tonic	10.9 ± 5.8	10.9 ± 6.6	9.7 ± 5.2	9.9 ± 5.7	10.8 ± 6.9
Vinegar beverage	9.7 ± 5.1	10.9 ± 6.7	9.9 ± 5.6	10.6 ± 5.3	10.7 ± 5.4
**Group 8 bodily fluid**					
Saliva^b^	NT^c^	9.7 ± 6.5	10.5 ± 7.3	11.0 ± 6.2	10.9 ± 6.3
Urine^b^	10.5 ± 6.2	10.9 ± 6.7	10.2 ± 7.2	10.2 ± 6.5	11.1 ± 5.5
**Group 9 viral transport media**					
PBS	11.2 ± 7.5	11.2 ± 6.8	10.0 ± 5.2	11.0 ± 6.7	10.9 ± 5.5
10% FBS/MEM	11.1 ± 7.1	11.7 ± 8.1	11.5 ± 7.8	11.0 ± 6.3	10.9 ± 5.5

*Viral genome levels are expressed as mean ± standard deviation log10 genome copies/mL. Genome level was determined by real-time PCR by using the E-gene for detection. All experiments were performed using at least two independent duplicates.*

*^a^Indicates alcohol content.*

*^*b*^Pooled saliva and urine were obtained from Innovative Research, Inc. (Novi, MI, United States). FBS/MEM, 10% fetal bovine serum with minimum essential medium; PBS, phosphate-buffered saline.*

*^c^NT indicates not tested.*

**TABLE 3:** Half-life value (Day) of SARS-CoV-2 spiked in different beverages and bodily fluids.

**Sample**	**Half-life value (Day)**
**Group 1 water**	
Mineral water	52.7
Tap water	15.1
Distilled water	25.7
**Group 2 milk**	
Milk	38.6
Soy milk	38.6
Yogurt drink	3.0
Milk-coffee	6.3
Cocoa	8.4
**Group 3 juice/coffee**	
Apple juice	0.7
Orange juice	4.7
Tomato juice	3.4
Flavored fruit juice	2.6
Black coffee	21
**Group 4 carbonated beverage**	
Cola	4.5
Lemon-lime carbonated beverage	4.0
**Group 5 tea**	
Green tea	1.2
Black tea (Oolong tea)	NT^c^
Herbal tea (*Artemisia capillaris*)	NT
**Group 6 alcohol bevarage**	
Beer, 5%^a^	9.2
Sparkling sake, 5%^a^	1.6
Carbonated distilled beverage (shochu), 9%^a^	14
White wine, 11%^a^	NT
Japanese wine (sake), 15.6%^a^	6.5
**Group 7 health beverage**	
Sports drink P	1.6
Amino sports beverage	0.5
Multivitamin beverage	1.6
Energy tonic	NT
Vinegar beverage	2.1
**Group 8 bodily fluid**	
Saliva^b^	4.0
Urine^b^	10.5
**Group 9 viral transport media**	
PBS	17.5
10% FBS/MEM	15.4

*The half-life value (day) of the infectious virus in each liquid sample was calculated using;*

*Half-life value (t 1/2) = log_10_2/k, k = decay constant.*

*^*a*^Indicates alcohol content.*

*^*b*^Pooled saliva and urine were obtained from Innovative Research, Inc. (Novi, MI, United States).*

*FBS/MEM, 10% fetal bovine serum with minimum essential medium; PBS, phosphate-buffered saline.*

*^c^NT indicates not tested.*

## Discussion

Our data showed that SARS-CoV-2 infectivity was stable in commonly consumed beverages and bodily fluids at optimal storage conditions. These results demonstrated virus stability even in beverages with significant alcohol content, such as Japanese wine sake (15.6% of alcohol content). While there has been little evidence of the isolation of SARS-CoV-2 from sewage water, infectious virus was detected in spiked tap water and bodily fluids up to 2-months after incubation in which their half-life values were between 4.0 and 52.7 days (Table 3), indicating prolonged virus stability in drinking water and possibly wastewater. Viral RNA could still be detected at high levels in samples that did not demonstrate infectivity, concurring with that of previous studies that viral RNA levels do not reflect infectivity. Of note, in a similar experiment using a dengue virus (Supplementary Table 1), no infectious particles were detected after inoculation in all of the spiked-alcoholic beverage samples. Infectious SARS-CoV-2 could still be detected for the length of the experiment, of up to 77 days, in common beverages, including water and milk. While SARS-CoV-2 suspended in water, milk, and soy milk remained infectious up to day 77 of refrigeration, infectious virus particles were not detected in acidic beverages (fruit juices, cola, vinegar drink, and white wine) by day 7 after refrigeration. Of note, infectious virus particles were not detected in tea beverages immediately after incubation, indicating the presence of active compounds that may hamper SARS-CoV-2 infectivity *in vitro*. In all, the study provided insights into SARS-CoV-2 infectivity in common commercial beverages, with the longest follow-up period to date being 77 days, and it also demonstrated that SARS-CoV-2 has great stability in a range of environmental conditions. While plaque assay is laborious and requires high-level biosafety containment (BSL-3 facility), additional means of monitoring, including quantification of viral proteins by mass spectrometry (Gouveia et al., 2020), may be useful as proxies for infectious virus particles, and will be complementary in monitoring virus infectivity and potential risks.

Although aerosol transmission remains the main route of transmission for SARS-CoV-2, other routes of transmission including surface contact and fecal–oral route could not be ruled out. In this context, the possible risk of Middle East respiratory syndrome (MERS) transmission in contaminated milk has been suggested previously (van Doremalen et al., 2014). While, SARS-CoV-2 RNA has been detected in beverages and biological samples including urine of some patients (Wang et al., 2020; Wu et al., 2020; Xiao et al., 2020), there is limited evidence of transmission by SARS-CoV-2 through food consumption or fecal–oral transmission (Xiao et al., 2020). As human gut enterocytes have been reported to be permissive to the replication of SARS-CoV-2 (Lamers et al., 2020), these studies imply that further studies would be needed to determine virus transmissibility through the fecal–oral route. Viral transmission may be affected by various environmental factors, including temperature, humidity, and route of transmission (Chin et al., 2020; Ratnesar-Shumate et al., 2020; Ren et al., 2020). Additionally, with the current industry standards for food manufacturing (Anelich, 2020), the risk of food contamination through exposure to contaminated products and contamination of the production line is likely low and thus, public health threat due to the consumption of contaminated food is likely limited. However, given that commercially produced beverages are often produced on a large-scale and exported to other countries, the finding of this study demonstrating that SARS-CoV-2 is stable in optimal conditions for more than 2 months indicates that a sufficient amount of infectious virus may potentially lead to virus transmission, and this points to possible exposure through the handling of contaminated food and beverages. Newer approaches and paradigm shift to the global food and beverage supply chain, including control measures including routine monitoring and disinfection, will be key in reducing the risk of exposure and in outbreak control and prevention.

## Data Availability Statement

The original contributions presented in the study are included in the article/Supplementary Material, further inquiries can be directed to the corresponding author/s.

## Author Contributions

MF, ZM, KM, and MLM contributed to the design and implementation of the research, to the analysis of the results, and to the writing of the manuscript. MF, ZM, and MLM carried out the experiments. KM and MLM contributed reagents to the study. MLM conceived the study and was in charge of overall direction and planning. All authors contributed to the article and approved the submitted version.

## Conflict of Interest

The authors declare that the research was conducted in the absence of any commercial or financial relationships that could be construed as a potential conflict of interest.
