# Supplementary material for: Stability and Infectivity of SARS-CoV-2 and Viral RNA in Water, Commercial Beverages, and Bodily Fluids
Source: Front Microbiol. 2021 May 5;12:667956. doi: 10.3389/fmicb.2021.667956 (PMC8131666; doi:10.3389/fmicb.2021.667956)
Supplement: Supplementary file 1 [file Data_Sheet_1.docx]

Table 1: Stability and infectivity of DENV-1 in different beverages and bodily fluids over a 28-day period

|  | **Virus titer (log10 PFU/mL)** | | | | | |
| --- | --- | --- | --- | --- | --- | --- |
| **Sample** | **Day 0** | **Day 3** | **Day 7** | **Day 14** | **Day 21** | **Day28** |
| Mineral water | ND^3^ | 3.5±0.3 | ND | ND | 3.7±0.4 | 3.5±0.1 |
| Milk | ND | 5.0±0.1 | 5.1±0.0 | 4.5±0.0 | 4.7±0.4 | 4.9±0.0 |
| Soy Milk | ND | 3.8±0.2 | 3.4±0.3 | ND | ND | ND |
| Apple juice | ND | ND | ND | ND | ND | ND |
| Orange juice | ND | ND | ND | ND | ND | ND |
| Tomato juice | ND | ND | ND | ND | ND | ND |
| Flavored fruit juice | ND | ND | ND | ND | ND | ND |
| Yogurt drink | ND | ND | ND | ND | ND | ND |
| Black coffee | ND | ND | ND | ND | ND | ND |
| Milk-coffee | ND | ND | ND | ND | ND | ND |
| Cocoa | ND | ND | ND | ND | ND | ND |
| Vinegar drink | ND | ND | ND | ND | ND | ND |
| Cola | ND | ND | ND | ND | ND | ND |
| Lemon-lime carbonated drink | ND | ND | ND | ND | ND | ND |
| Green tea | ND | ND | ND | ND | ND | ND |
| Black tea (Oolong tea) | ND | ND | ND | ND | ND | ND |
| Herbal tea (*Artemisia capillaris*) | ND | ND | ND | ND | ND | ND |
| Beer, 5%^1^ | ND | ND | ND | ND | ND | ND |
| Sparkling sake, 5%^1^ | ND | ND | ND | ND | ND | ND |
| Carbonated distilled beverage (shochu), 9%^1^ | ND | ND | ND | ND | ND | ND |
| White wine, 11%^1^ | ND | ND | ND | ND | ND | ND |
| Japanese wine (sake), 15∙6%^1^ | ND | ND | ND | ND | ND | ND |
| Sports drink P | ND | ND | ND | ND | ND | ND |
| Amino sports drink | ND | ND | ND | ND | ND | ND |
| Multivitamin drink | ND | ND | ND | ND | ND | ND |
| Energy tonic | ND | ND | ND | ND | ND | ND |
| Saliva^2^ | ND | ND | ND | ND | ND | ND |
| Urine^2^ | ND | ND | ND | ND | ND | ND |
| Distilled water | ND | 4.2±0.1 | 2.7^4^ | ND | 3.6±0.5 | 4.5±0.2 |
| PBS | ND | 4.0±0.1 | ND | ND | NT^5^ | 3.7±0.2 |
| 10% FBS/MEM | ND | 4.5±0.1 | 4.7±0.0 | 4.2±0.0 | 3.1±0.0 | 3.5±0.0 |

Viral titers are expressed as mean ± standard deviation log10 PFU/mL. Viral titers were titrated using Vero cells. All experiments were performed using at least two independent duplicates.

^1^Indicates alcohol content.

^2^Pooled saliva and urine was obtained from Innovative Research, Inc. (Novi, MI, USA).

^3^ ND below detection limit. The detection limit of the assay was 100 PFU/mL.

^4^ Only one replicate demonstrated plaques.

^5^ NT indicates not tested

FBS/MEM, 10% fetal bovine serum with minimum essential medium; PBS, phosphate-buffered saline
